# ‘Erring on the side of rare events’? A behavioural explanation for COVID-19 vaccine regulatory misalignment

**DOI:** 10.7189/jogh.11.03080

**Published:** 2021-07-10

**Authors:** Joan Costa-Font, Miqdad Asaria, Elias Mossialos

**Affiliations:** 1 Department of Health policy, London School of Economics and Political Science, London, UK; 2 Centre for Health Policy, Imperial College London, London, UK

The development of new vaccines against COVID-19 has triggered a debate about which of the vaccines should be chosen, and in some countries which vaccines to authorise. The choice of vaccines in Western countries seems to be largely driven by trust in the vaccine manufacturers, with safety concerns regarding potential rare side effects rather than relative efficacy playing the pivotal role in this choice [1]. So far, vaccines developed by China, Russia and India have largely been ignored in Western countries. Amongst the vaccine candidates currently in use in many western countries, access to the AstraZeneca (AZ) and Johnson & Johnson’s (JJ) vaccine has been restricted and, in some cases, suspended as they hav been perceived to be the least safe despite being approved by the European Medicines Agency (EMA) [2] and recommended by the WHO [3].

We argue that regulatory vaccine misalignment can be explained by an ‘erring on the side of rare events’ phenomena. That is, when rare events are heavily publicised, regulators tend to favour a precautionary approach, even when the fatalities from vaccine side effects are only 10% as likely to occur as the risks arising from COVID-19 infection [3]. Furthermore, we argue that such decisions have detrimental consequences for vaccine trust and the success of vaccination programmes globally. Such behavioural phenomena logically follow not just from the overestimation of the risk of such events, but also from a combination of ambiguity aversion, joint risk, and benefit formation. All of which add to a background of limited trust in government decisions with regards to vaccines, which weaken the vaccination rollout. This note will provide a discussion of these arguments.

The next section argues that vaccine regulation follows a clear regulatory misalignment resulting from some countries ‘erring on the side of rare events. We examine the erosion of public trust, followed by a discussion on different behavioural explanations for the ‘erring on the side of rare events’ phenomena. Finally, we conclude with suggestions for a way forward.

## REGULATORY VACCINE MISALIGNMENT

Regulators across western countries have demonstrated significantly heterogeneous criteria in their covid-19 vaccine authorisation decisions. The UK has championed the AZ vaccine administering more than 20 million first doses by the end of March 2021 [4]. The UK regulator – the Medicines and Healthcare products Regulatory Agency (MHRA) – has identified 168 cases and 32 deaths of AZ vaccine recipients experiencing rare blood clots. All except one of the rare clots case reports came after a first dose. A risk far outweighed by the benefit derived from vaccination in terms of protection from Covid-19 particularly in older recipients Similarly, the EMA has regarded the AZ vaccine as ‘safe’, as its benefits outweigh its risks [2]. However, several countries around the world have restricted access to AZ vaccines particularly in younger population groups.

**Figure:**
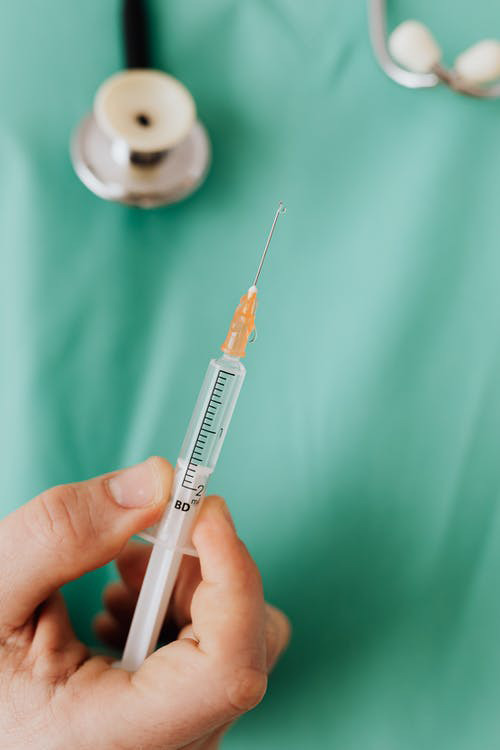
Photo: By Karolina Grabowska from Pexels.

Nonetheless, most EU countries have now applied age restrictions to the rollout of the AstraZeneca jab, despite it receiving regulatory approval for use in all people over the age of 18. Prior to the current controversy, 10 European countries had restricted use of the AZ vaccine in over 65s due to lack of data from Oxford’s Phase III trial, despite the EMA recommendations to continue to vaccinate this population. Some countries, such as Denmark, have even announced pulling the AZ vaccine from their vaccination programme outright. The way in which the different countries have responded to the AZ vaccine demonstrates the heterogeneity in regulatory policies across Europe - many of which it can be argued are driven by regulatory over-reaction. Similar decisions have been made with regards to Johnson and Johnson (JJ) vaccine.

## EROSION OF PUBLIC TRUST

The AZ vaccine got off to a bad start with the publication of confusing early trial data eroding public confidence in the vaccine. Additional safety concerns were raised about the use of the vaccine in the elderly population once it was understood that only 10 percent of those in the vaccine trial were aged 65 or above. Further controversy followed when regulators announced that AZ had omitted data from their trial results that may have impacted efficacy estimates [5]. Most recently, concerns have been raised that the vaccine may be associated with very rare cases of potentially fatal blood clots associated with thrombocytopenia. Finally, in some countries such as South Africa the AZ vaccine has been shown to have limited efficacy against the dominant variant of the virus circulating there and so has been discontinued raising further concerns in other countries.

A recent YouGov poll reports that 55% of Germans and 61% of French people now see the AZ vaccine as unsafe [6]. We discuss how such risks perceptions deriving from seemingly modest risk can be explained and what the implications are for vaccination programs globally. In the US, only 38% of Americans consider the AstraZeneca vaccine safe, while 27% believe it to be unsafe and a further 35% are unsure. Similarly, the Centre for Disease Control recommendation to pause Johnson & Johnson vaccine use caused public confidence in the vaccine to sink 15pts from 52% to 37% as reported in a subsequent YouGov poll [6].

## BEHAVIOURAL EXPLANATIONS FOR ‘ERRING ON THE SIDE OF RARE EVENTS’

The erosion of public trust resulting from regulatory misalignment can be explained by the combined effects of some well-known phenomena such as the overestimation of small and rare events, ambiguity aversion resulting from conflicting regulatory signals from different countries and limited perception of benefit.

### Overestimation of low probability, heavily publicised and very rare events

**Figure 1:**
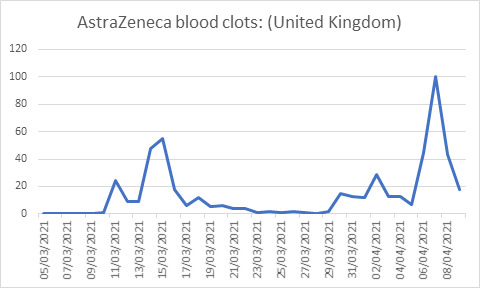
Google Trends search for AstraZeneca’s blood clots in the UK. Source: google trends.

The WHO’s 3 Cs model capturing the roles of confidence, complacency and convenience is a useful way to think about attitudes to vaccines [7]. Vaccine uptake is negatively impacted by a reduction in confidence, an increase in complacency and with increasing inconvenience. We relate these factors to various behavioural theories to understand the public and policy makers’ responses to the AZ vaccine. Although side effects rates of fewer than one in 10 000-100 000 are generally defined as ’very low’, they are subject to the well-known overestimation of small probability bias . The exaggeration of the side effects of the AZ vaccine is likely subject to this bias arising from the so-called availability heuristic (what is more publicised is more salient). When a rare event is very salient it is more likely to be retained in people’s memory and the subjective probability (risk perception) exceeds the objective probability. Similar over-estimation is observed when people are asked to judge the likelihood of rare but dramatic causes of death [8]. Such overestimates of risk of side effects disproportionately undermine confidence in the vaccine. **Figure 1** describes the changes in interest in AZ blood clots over time with the increasing publicity of the small probability of rare side effects.

### Ambiguity aversion

Heterogeneous decisions by different governments give rise to misunderstandings that might have an impact on people’s perceptions of vaccine effectiveness and safety. This operates through the presence of ambiguity aversion, or the presence of known uncertainty which has already been documented to lead people to avoid certain medical treatments [9]. Such ambiguity undermines confidence in the policy makers rolling out the vaccine programs. Ambiguity suggests that there is a role for improving the way risks are communicated. The Winton Centre for Risk and Evidence Communications have compiled a comparison of the risks and harms arising from the AZ vaccine as compared to other risks reproduced in **Table 1** below. Adverse side-effects of medications that occur in more than one in 10 000 (100 per million) are typically included on the label.

**Table 1:** Side effects of AZ compared to other risks*

	Age: 25	Age: 55
Serious harm from AZ vaccine side effects	11 per million	4 per million
Death from COVID-19	23 per million	800 per million
Death from accidental injury	110 per million	180 per million
Death from road accident	38 per million	23 per million
Being hit by lightning this year	1 per million	1 per million

AZ – AstraZeneca

*Source: Winton Centre for Risk Communication, 2021 [10].

### Joint risk benefit decision making

Another explanation lies in the idea that people require a much larger perceived benefit to outweigh a small perceived loss [11]. In a setting of relative certainty and comfort, small potential losses resulting from protective actions such as the take up of a vaccine must be outweighed by relatively large perceived benefits derived from vaccination [12]. This ‘omission bias’ is particularly relevant for younger population groups who are at low risk from Covid-19 mortality especially in settings where overall deaths from the virus are waning resulting in complacency increasing whilst confidence simultaneously falls. Vaccination in these groups can be perceived as higher risk than the reversible decision to not take the vaccine.

## THE WAY AHEAD

Misaligned regulatory decisions resulting from different countries ‘erring on the side of rare events’ to differing degrees, play a crucial role in eroding peoples’ trust and exaggerating their perception of risk’ thereby hampering the relative value of vaccines. This role of trust is also demonstrated in the general perceptions of vaccines produced by untrusted countries and vaccine hesitancy in marginalised communities that have low levels of trust in their governments such as ethnic minority communities in the UK [13]. Addressing these deficits of trust is likely the key factor underpinning the success of any vaccination program. A key lesson is that decisions regarding vaccines should be discussed and aligned across countries to avoid the perceived ambiguity that arises from heterogeneous regulation and feeds mistrust.

Regulatory decisions such as those regarding the AZ vaccine and the publicity surrounding them can easily become politicised. This may have pushed decision makers to adopt extreme forms of ‘erring on the side of rare side effects’ rather than be guided by objective data on risks. Instead of this regulatory over-reaction, a better response may be to communicate risks of unfamiliar and rare events to people in ways that are easily understandable and comparable to events they are familiar with. Presenting the levels of risk of rare side-effects occurring with and without vaccination as well as the difference in these risks across different vaccines may help contextualise such risks.

It is likely that the drop in confidence associated with the controversy around the AZ vaccine will have far reaching consequences for the vaccination program overall – with the public becoming more skeptical of all COVID-19 vaccines. Furthermore, many of the other vaccine candidates require highly specialised infrastructure such as extreme cold chains making it likely that they will be able to be delivered at many fewer suitably equipped locations. This will greatly reduce the access to and convenience of vaccinations even in the high-income countries where such alternatives are available. And finally, as many of the older and highest risk patients get vaccinated, falling hospitalisation and death rates from COVID-19 will increase the complacency in those who remain unvaccinated. None of these bode well for the next stages of the vaccination program.
